# Secretome analysis of *Strongyloides venezuelensis* parasitic stages reveals that soluble and insoluble proteins are involved in its parasitism

**DOI:** 10.1186/s13071-018-3266-x

**Published:** 2019-01-09

**Authors:** Yasunobu Maeda, Juan Emilio Palomares-Rius, Akina Hino, Tanzila Afrin, Shakhinur Islam Mondal, Ayako Nakatake, Haruhiko Maruyama, Taisei Kikuchi

**Affiliations:** 1 Division of Parasitology, Faculty of Medicine, University of Miyazaki, Miyazaki, 889-1692 Japan; 2 Instituto de Agricultura Sostenible, Consejo Superior de Investigaciones Científicas (CSIC), Avda. Menéndez Pidal s/n, 14004 Córdoba, Spain; 3 Department of Environmental Parasitology, Tokyo Medical and Dental University, Yushima, Bunkyo-ku, Tokyo, Japan; 4 HTLV-1/ATL Research Facility, Faculty of Medicine, University of Miyazaki, Miyazaki, 889-1692 Japan

**Keywords:** Animal parasitic nematode, Secretome, Adhesives, Histones, Trypsin inhibitor-like

## Abstract

**Background:**

Parasites excrete and secrete a wide range of molecules that act as the primary interface with their hosts and play critical roles in establishing parasitism during different stages of infection. *Strongyloides venezuelensis* is a gastrointestinal parasite of rats that is widely used as a laboratory model and is known to produce both soluble and insoluble (adhesive) secretions during its parasitic stages. However, little is known about the constituents of these secretions.

**Results:**

Using mass spectrometry, we identified 436 proteins from the infective third-stage larvae (iL3s) and 196 proteins from the parasitic females of *S. venezuelensis*. The proteins that were secreted by the iL3s were enriched with peptidase activity, embryo development and the oxidation-reduction process, while those of the parasitic females were associated with glycolysis, DNA binding (histones) and other unknown functions. Trypsin inhibitor-like domain-containing proteins were identified as the main component of the adhesive secretion from parasitic females. An absence of secretion signals in many of the proteins indicated that they are secreted *via* non-classical secretion pathways.

**Conclusions:**

We found that *S. venezuelensis* secretes a wide range of proteins to establish parasitism. This includes proteins that have previously been identified as being involved in parasitism in other helminths as well as proteins that are unique to this species. These findings provide insights into the molecular mechanisms underlying *Strongyloides* parasitism.

**Electronic supplementary material:**

The online version of this article (10.1186/s13071-018-3266-x) contains supplementary material, which is available to authorized users.

## Background

**Fig. 1:**
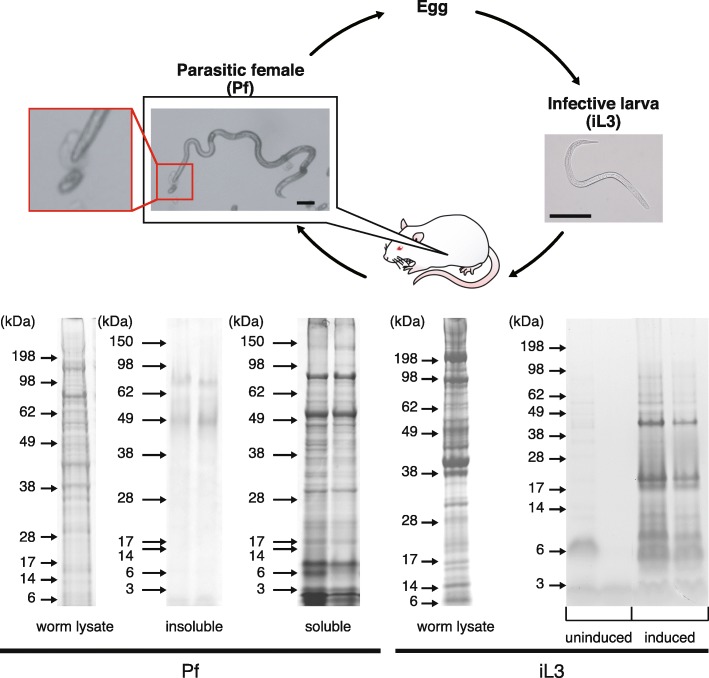
Simplified life-cycle of *Strongyloides venezuelensis* and sodium dodecyl sulphate polyacrylamide gel electrophoresis (SDS-PAGE) gel images of excretory/secretory (E/S) proteins from parasitic females (Pfs) and infective third-stage larvae (iL3s). During the life-cycle of this species, the iL3 infects the host percutaneously and then migrates through the body to the small intestine, where it develops into an adult parasitic female that produces eggs parthenogenetically. The parasitic female secretes adhesive substances from the mouth, as shown in the inset picture. Parasitic female secretions were collected into two fractions (soluble and insoluble adhesive substances). *Scale-bars*: 100 μm

The genus *Strongyloides* comprises more than 50 species of nematodes that parasitise amphibians, reptiles, birds and mammals [1]. Two species, *S. sterocoralis* and *S. fuelleborni*, are parasites of humans, infecting approximately 200 million people worldwide [2–4]. *Strongyloides* nematodes have a complex but interesting life-cycle. Infection begins when the infective third-stage larvae (iL3s) that inhabit soil/faeces penetrate the host’s skin. After entering the host, they migrate through the host’s body to their final destination, the small intestine, where they moult twice to develop into parasitic adults. The parasitic adults, which are all female, then produce eggs by parthenogenesis. Once the eggs or hatched larvae are excreted from the host, they develop into iL3s (Fig. 1) [1].

*Strongyloides venezuelensis* is a gastrointestinal parasite of rodent species, particularly rats, and is found worldwide [5–7]. Alongside *S. ratti*, it is one of the most widely used laboratory models for studying *Strongyloides* infection and mucosal immunity [8–10]. Although both species parasitise rodents, it has been suggested that they use different strategies to establish parasitism, including migration routes within the host and the mechanisms of host immunomodulation that allow successful parasitism [8]. Furthermore, they also differ in their reproductive strategies, gonad structures, karyotypes and phylogenetic positions within the genus, suggesting that they evolved into parasites of rodents independently [8, 11].

Parasites produce a wide range of excretory/secretory (E/S) components that act as the primary interface with their hosts [12]. These proteins and other molecules are likely to play critical roles in establishing parasitism during all stages of infection, including recognition/invasion of the host and immune evasion [13, 14], making them promising targets for drugs or vaccines. Consequently, E/S components have been analysed in a wide range of parasitic nematodes, such as *S. ratti* [15], *S. stercoralis* [16], *Ascaris suum* [17], *Brugia malayi* [13], *Ancylostoma caninum* [12] and *Trichinella spiralis* [18].

The advancement of mass spectrometry and associated technologies has accelerated research in this field by allowing the rapid and sensitive identification of proteins in E/S components. A previous proteomic analysis of *S. ratti* identified a variety of E/S proteins that were produced by parasitic females and iL3s, including peptidases, lectins, anti-oxidative enzymes, heat shock proteins and carbohydrate-binding proteins [15]. Comparison of the E/S components of *S. ratti* and *S. venezuelensis* would help us to understand the molecular mechanisms underlying their modes of parasitism. Therefore, in this study we performed a proteomic analysis of *S. venezuelensis* E/S components using mass spectrometry combined with the recently established high-quality reference genome. Parasitic female *S. venezuelensis* secrete adhesive molecules that form insoluble complex secretions [19], which are likely to be important for their attachment to the intestinal epithelium and the construction of the walls of worm tunnels in the mucosal epithelial layer in which they lodge themselves [20]. Therefore, we analysed both the hydro-soluble components and the insoluble adhesives that are secreted by *S. venezuelensis*.

## Methods

### Parasites and host animals

The *S. venezuelensis* HH1 isolate [5] that was used in this study had been maintained in the Parasitology Laboratory of the University of Miyazaki, Japan, by serial passage in male Wistar rats purchased from Kyudo Co. Ltd. (Kumamoto, Japan). Infectious aliquots were prepared by faecal culture using filter paper at 27 °C for 2 days, following the methodology of Hino et al. [11]. The nematodes were then washed three times in distilled water and approximately 30,000 nematodes were administered by subcutaneous injection.

### Preparation of E/S components of iL3s

Live iL3 nematodes were isolated from a faecal culture using the filter paper method and were washed five times in sterile phosphate-buffered saline (PBS) to remove any debris. Approximately 400,000 nematodes were preincubated in 65 ml of PBS with antibiotics [100 U/ml of streptomycin (Sigma-Aldrich, Tokyo, Japan) and 30 μg/ml of chloramphenicol (Sigma-Aldrich)] for 12 h at 37 °C under 5% CO_2_ (uninduced condition). The nematodes were then transferred to 65 ml of high-glucose Dulbecco’s Modified Eagle Medium (DMEM; 4.5 g/l of D-glucose + L-glutamine; Life Technologies, Tokyo, Japan) supplemented with antibiotics [100 U/ml of penicillin Sigma-Aldrich), 100 U/ml of streptomycin (Sigma-Aldrich) and 30 mg/ml of chloramphenicol] and incubated at 37 °C under 5% CO_2_ to induce secretion (induced condition). To collect E/S proteins, worms were incubated for 36 h in the medium (*nota bene*, worms incubated under the uninduced condition continuously in PBS at 37 °C start dying after 12 h). After incubation, the cultures were viewed under a microscope and only those that contained viable and sterile nematodes were used in the analyses. The incubated solutions (PBS for 12 h or DMEM for 36 h for uninduced and induced samples, respectively) were concentrated to approximately 100 times using a microconcentrator with a 10-kDa molecular weight cutoff (Amicon Ultra-15; Millipore, Tokyo, Japan) and all remaining nematodes were homogenised by a polytron (Kinematica Polytron, Model PT 3000, Luzern, Switzerland) and used as worm lysate.

### Preparation of E/S components from parasitic females

Parasitic females were freshly collected from rat intestines at 6 days post-infection (dpi), as described previously [11], and were filtered through a sieve (ø 25 μm) and washed three times with PBS to remove any residual proteins or host tissues. The cleaned nematodes (*n* = 6000–10,000) were then transferred to a plastic dish (ø 35 mm) and incubated in sterilised PBS with antibiotics [100 U/ml of penicillin (Sigma-Aldrich), 100 U/ml of streptomycin (Sigma-Aldrich) and 30 μg/ml of chloramphenicol (Sigma-Aldrich)] at 37 °C or 4 °C for 24 h. For the proteinase inhibitor tests, protease inhibitor cocktail [one tablet of cOmplete™, Mini Protease Inhibitor Cocktail (Roche Diagnostics, Mannheim, Germany) dissolved in 17.5 ml of water] was added to the incubation solution. After incubation, the hydro-soluble E/S products (culture supernatant) were concentrated to approximately 100 times using a microconcentrator with a 10-kDa molecular weight cutoff (Amicon Ultra-15 centrifugal filter units; Millipore). After thoroughly washing the plastic dish with PBS to remove all of the adult worms and eggs, adhesion spots on the dish surface were solubilised with guanidine-HCl buffer [8 M guanidine, 50 mM Tris-HCl (pH 8.0), 50 mM dithiothreitol (DTT)] and collected as the hydro-insoluble sample. All remaining nematodes were homogenised by a polytron (Kinematica Polytron, Model PT 3000) and used as worm lysate.

### One-dimensional electrophoresis and tryptic digestion of E/S proteins

Protein samples that had been concentrated by microconcentrators (soluble proteins) or dissolved in guanidine-HCl buffer were incubated overnight at -30 °C and then precipitated with ethanol (90% at final concentration) by centrifugation at 12,000× *g* for 10 min. The precipitated proteins were dissolved in sodium dodecyl sulphate (SDS) loading buffer [1% SDS, 0.375 M Tris-HCl (pH 8.8), 50 mM DTT, 25% (v/v) glycerol and 0.05% bromophenol blue] and heat-denatured for 10 min at 70 °C. The protein concentrations were measured using a Quant-iT™ Protein Assay Kit and a Qubit fluorometer (Invitrogen, Tokyo, Japan) according to the manufacturer’s instructions. Approximately 15 μg of protein per sample was loaded on a 10% sodium dodecyl sulphate polyacrylamide gel electrophoresis (SDS-PAGE) gel (NuPAGE Novex, Invitrogen) with 10 μl of SeeBlue® Plus2 Pre-stained Standard (Invitrogen) as a protein weight marker. Electrophoresis was performed using a maximum of 200 V for 30 min, following which the gels were stained with Coomassie Brilliant Blue G-250 (Bio-Rad, Hercules, USA). The lanes containing proteins were sliced into small fragments using disposable scalpels. Clear bands were preferentially cut into thin slices while the remainder were sliced roughly into 2-cm-wide strips (*n* = 40–50 pieces per sample). The gel pieces were then washed for 5 min in 0.1 M ammonium bicarbonate, de-stained in 50 mM ammonium bicarbonate buffer containing 1/3 acetonitrile for 60 min and dehydrated in 100% acetonitrile.

For in-gel tryptic digestion, the gel pieces were hydrated for 45 min in sequencing grade trypsin solution (12.5 ng/l; Promega, Madison, USA) on ice. The remaining trypsin solution was removed and the gel pieces were covered with 50 mM ammonium bicarbonate and incubated overnight at 37 °C, following which the supernatants containing the majority of the tryptic peptides were transferred into new 1.5-ml plastic tubes. The gel pieces were then washed in ammonium bicarbonate containing 1/3 acetonitrile and the washing buffer was pooled with the respective supernatant. Finally, any remaining peptides were extracted with 100% acetonitrile and added to the pooled supernatant and washing solution.

### Mass spectrometric analysis

The peptides that were derived from the in-gel digested proteins were analysed by liquid chromatography-mass spectrometry (LC-MS) using a DiNa nanoLC system (KYA Technologies, Tokyo, Japan) coupled online to a LCMS-IT-TOF mass spectrometer (Shimadzu Scientific Instruments, Kyoto, Japan). The LC separation proceeded using a PicoFrit column BetaBasic C18 (New Objective, Woburn, USA) at a constant flow rate of 300 nl/min. Peptides were eluted from 10 μl samples using gradients of 2–50% solvent B (0.1% formic acid in 95% ACN)/0–25 min, 50–100% solvent B/25–28 min, and 100% solvent B/28–35 min. LCMS-IT-TOF was operated in the data-dependent MS/MS mode. The capillary temperature and electrospray voltage were set at 200 °C and 1.8 kV, respectively. Data were collected at scan ranges of mass/charge (m/z) 400–1500 for MS and m/z 50–1500 for MS/MS. Proteins were considered to be present when they were found in at least one of the replicates. LCD files were generated by LCMS solution software v.3 and converted into MGF files using Mascot Distiller v.2.4.3.3.

### Protein identification and functional annotation

Proteins were identified by automated database searching (Mascot Daemon v.2.4.0; Matrix Science, London, UK) against Swiss-Prot (547,357 proteins) or the *S. venezuelensis* genome local database (v.2.0; 18,048 proteins) [21] using the following parameters: enzyme, trypsin; number of missed cleavages permitted, 1; fixed modifications, carbamidomethylation of cysteines; variable modifications, oxidation of methionine; peptide charge, +1, +2 and +3; peptide tolerance, ± 1.2 Da; MS/MS tolerance, ± 0.6 Da; instrument, ESI-TRAP. We used the MASCOT “contaminants” option and the cRAP database (https://www.thegpm.org/crap/) to remove possible contaminants. To assess the incidence of false positive identifications, the data were also searched against an inverted tryptic peptide database (decoy database) and a false discovery rate (FDR) of < 0.05 was used as a cutoff score for the protein identifications.

Functional annotation of the identified proteins was performed using several bioinformatic tools. Protein Basic Local Alignment Search Tool (BLASTP) searches were performed against the National Center for Biotechnology Information (NCBI) non-redundant protein (nr) database and an in-house nematode genome database that was constructed with protein sequence data obtained from the WormBase website (http://www.wormbase.org; release WS245). InterProScan was also used to search against the InterPro protein family database, which included PROSITE, PRINTS, Pfam, ProDom, Simple Modular Architecture Research Tool (SMART), TIGRFAMs, PIR SuperFamily (PIRSF), SUPERFAMILY, Signal Peptide (SignalP) and Transmembrane Helices Hidden Markov Models (TM-HMM) [22]. The BLAST results against the NCBI nr and InterProScan results were then loaded into Blast2GO v.2.7.2 [23] to annotate the sequences with gene ontology (GO) terms. GO enrichment analyses were performed with the whole *S. venezuelensis* proteins (18,048) as a reference using Fisher’s exact test with multiple testing correction (false discovery rate, FDR) implemented in Blast2GO software. A Pfam protein domain search (v.31.0) using HMMscan [24] was also performed independently from the InterProScan search with an e-value cutoff of 1.0. The NetOGlyc 4.0 server [25] was used for mucin type O-glycosylation site prediction.

### RNA extraction, library preparation and sequencing

iL3 nematodes were collected as described above for the preparation of E/S components. Uninduced iL3 worms were prepared by washing isolated iL3s with PBS five times. Induced iL3s were generated *in vitro* by incubating iL3 nematodes in DMEM (4.5 g/l of D-glucose + L-glutamine; Life Technologies) supplemented with antibiotics (0.25 mg/ml of gentamicin; Life Technologies) at 37 °C under 5% CO_2_ and worms were collected on 1 or 5 days of incubation. Parasitic gravid females were collected from rat intestines at 7 dpi, washed three times in PBS and immediately stored in 250 μl of TRI Reagent® at -80 °C prior to RNA extraction.

Total RNA was extracted from *S. venezuelensis* using TRI Reagent, according to the manufacturer’s instructions. RNA libraries were prepared with a TruSeq Stranded RNA Sample Prep Kit (Illumina, Little Chesterford, UK) and were sequenced on a HiSeq2000 sequencer (Illumina) following the manufacturer’s recommended protocol to produce 100-bp paired-end reads.

RNAseq reads were mapped to the *S. venezuelensis* reference genome (v.2.0) using TopHat2 (v.2.0.12) with the following options: --read-mismatches 2; --max-multihits 20 -a 8; --microexon-search; --min-segment-intron 35; --max-segment-intron 100000 -r 50. The Cufflinks package (v.2.2.1) was then used to obtain fragments per kilobase of exon per million reads mapped (FPKM) values as a normalised unit of gene expression in the RNAseq analysis [26].

Correlation analysis between the protein and gene expression levels was performed by comparing the exponentially modified protein abundance index (emPAI), which estimates the actual amount of a given protein in a protein mixture [27], with the corresponding FPKM values using Spearman’s rank correlation test implemented in R package v.3.1.1 (http://www.r-project.org).

## Results and discussion

### E/S sample preparation

E/S samples were prepared from two parasitic stages of *S. venezuelensis*: iL3s and parasitic females. We obtained approximately 40 μg of proteins from iL3s that were incubated in a nutrient-rich medium that simulated the conditions in the host animal (induced iL3 samples) but only a small amount of proteins from iL3s that were incubated in PBS (uninduced iL3 samples). Proteins from the induced-iL3 samples had molecular sizes ranging from 3 to 150 kDa, with approximately 30 visible bands in the SDS-PAGE gel (Fig. 1), whereas only weaker bands were seen in the uninduced samples (Additional file 1: Figure S1). The E/S protein band pattern was visibly different from somatic protein samples that were prepared from the body lysates of squashed nematodes (Additional file 1: Figure S1) and three biological replicates of each treatment showed similar patterns (data not shown). Therefore, it appears that iL3s secrete small amount of proteins while inhabiting the soil or faeces but initiate vigorous secretion once they encounter a host.

Parasitic female E/S products were collected from two separate fractions: a hydro-soluble fraction that was collected from the PBS in which nematodes had been incubated and a hydro-insoluble sample that was collected from the large number of adhesion spots that were observed on the surface of the Petri dish (Fig. 1). Approximately 60 and 30 μg of proteins were obtained from the hydro-soluble and -insoluble fractions, respectively. The hydro-soluble fraction contained proteins of a wide molecular size range that made up approximately 40 visible protein bands (Fig. 1), whereas the hydro-insoluble fraction yielded a few major bands in the range of 45–90 kDa, all of which were smearing in the gel (Fig. 1). These E/S patterns differed from those observed for the somatic protein samples, as well as from those of E/S samples that were prepared following incubation at 4 °C (Additional file 1: Figure S1), suggesting that the obtained samples mainly contained proteins that were secreted when the nematodes were active.

It is generally recommended that protease inhibitors are used when collecting proteins to minimise protein degradation. However, we found that iL3 nematodes that were incubated in DMEM with protease inhibitors with broad inhibitory specificity showed different morphologies from those that were incubated without protease inhibitors or were isolated from a rat lung (Additional file 1: Figure S2), indicating that protease inhibitors may inhibit the normal development of iL3s. Therefore, here, we used iL3 samples that had been prepared in the absence of proteinase inhibitors. About 30 visible bands in the SDS-PAGE gels were conserved between the absence and presence of proteinase inhibitors even with slight differences of the band strength between replicates (Additional file 1: Figure S1). The only big visible difference in the SDS-PAGE gel was found in around 6 kDa, in which we found a strong protein band in proteinase-inhibitor-presence samples only. We also avoided using proteinase inhibitors for the parasitic females because the ~40 visible bands were shared by the two conditions (Additional file 1: Figure S1b). Accordingly, we assume that presence/absence of proteinase inhibitors during the sample preparation did not significantly affect protein identification, although we might have missed small peptides in proteinase-inhibitor-absence samples, which are possibly caused by physiological differences of the worms between the two conditions.

### Protein identification

**Fig. 2:**
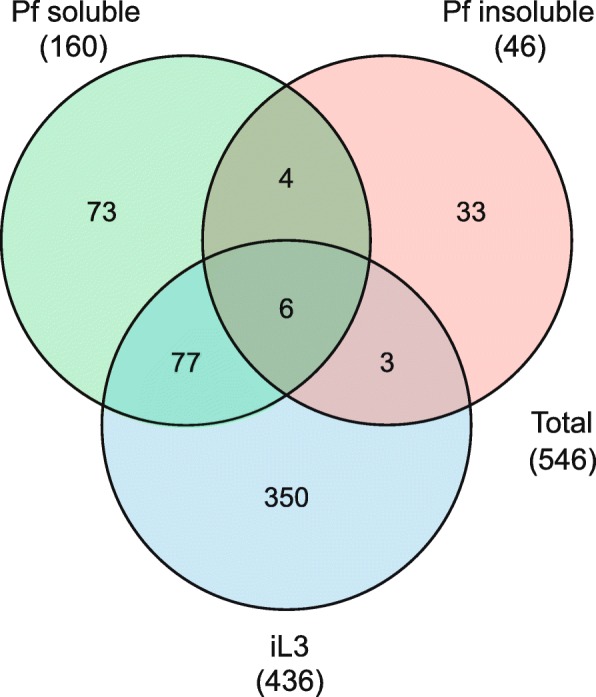
Venn diagram showing the number of identified proteins in infective larvae (iL3s), the parasitic female (Pf) soluble fraction and the Pf insoluble fraction of *Strongyloides venezuelensis*

The mass spectrometry analyses identified 436 proteins from the iL3s, 160 proteins from the hydro-soluble fraction of parasitic females and 46 proteins from the hydro-insoluble fraction of parasitic females, representing a total of 546 different proteins (Additional file 2: Table S1, Additional file 3: Table S2, Additional file 4: Table S3). The lengths of these proteins ranged from 66 to 8239 amino acids (mean = 661). Among the identified proteins, 350 were specific to iL3s, 73 to the hydro-soluble fraction of parasitic females and 33 to the hydro-insoluble fraction of parasitic females (Fig. 2). A total of 486 (89.0%) the proteins showed high similarity to proteins in the NCBI nr database (BLASTP, e-value < 1e-20) and 446 (81.7%) showed at least one Pfam protein domain match. Furthermore, 434 (79.5%) of the sequences were annotated with at least one GO term. The assigned GO terms spanned a wide range of categories but the terms ‘catalytic activity’ and ‘binding’ accounted for 90% of all assigned terms in all three sample groups, suggesting that these functions play an important role in the E/S products of this parasite (Additional file 2: Table S1, Additional file 3: Table S2, Additional file 4: Table S3). In addition, the proteins that were identified in the parasitic females were enriched with the GO terms ‘glycolysis’, ‘protein folding’, ‘DNA binding complex’ and ‘oxidation-reduction process’ (Additional file 1: Table S4), whereas iL3 proteins were enriched with a range of GO terms, including ‘embryo development’, ‘glycolysis’ and ‘receptor mediated endocytosis’ (Additional file 1: Table S5).

The SignalP analysis detected eukaryotic signal peptides in 110 (20.1%) of the 546 proteins (Additional file 2: Table S1, Additional file 3: Table S2, Additional file 4: Table S3). Therefore, considering that these proteins were collected from outside the nematode body, many of the proteins that were identified can evidently be excreted/secreted *via* ‘non-classical pathway’ that does not require signal peptides, such as direct translocation from cytoplasm across the plasma membrane through membrane transporters, lysosomal secretion, blebbing and release *via* exosomes [28, 29].

### iL3 proteins

**Fig. 3:**
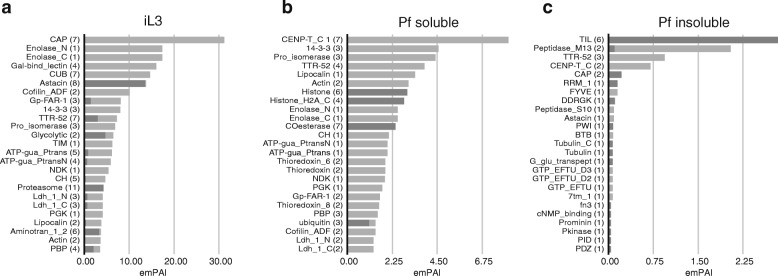
Top 25 Pfam protein domains identified in infective third-stage larvae (iL3s) (**a**), the parasitic female (Pf) soluble fraction (**b**) and the Pf insoluble fraction of *Strongyloides venezuelensis* (**c**). The sum of the exponentially modified protein abundance index (emPAI) values of identified proteins was calculated for each Pfam domain and the 25 most abundant terms were listed. The amounts of proteins that were specifically detected in each sample group are shown in shaded gray. Numbers in parentheses show the numbers of proteins with corresponding Pfam domains

Of the 436 proteins that were identified in the iL3 samples, 350 were specific to iL3s (Fig. 2). Protein domain analysis revealed that these proteins contained a wide range of Pfam domains (Fig. 3a). We found many proteolysis-related domains, such as astacin, CUB and proteasome, with astacin-like peptidases in particular having high emPAI values (Additional file 2: Table S1), suggesting that they play an important role in the infective stage. Astacin-like peptidases are widely distributed in animal-parasitic nematodes, including *Strongyloides* spp. [15, 21], *Ancylostoma* spp. [30] and *Onchocerca* spp. [31], and are thought to be involved in invasion and migration in the host’s body [32]. In addition, three of the top 20 proteins contained CAP domains (cysteine-rich secretory proteins, antigen 5 and pathogenesis-related 1 proteins). The function of CAP proteins, which are also known as sperm-coating-protein/Tpx-1/Ag5/PR-1/Sc7 (SCP/TAPS) proteins, is currently unclear but it has been suggested that they are involved in immunomodulation and host-parasite interactions in parasitic helminths [33]. In *Ancylostoma caninum*, an abundance of SCP/TAPS proteins was identified in the E/S products of iL3s, which were proposed to be important for the transition to parasitism, such as the transition from the ensheathed free-living stage to the activated third-stage larvae [34], invasion of the mammalian host [35] and the response to host-specific signals during the infection process [36, 37]. The high abundance of SCP/TAPS proteins in *S. venezuelensis* iL3 E/S products suggests that they may play similar roles in this parasite.

Proteins that were specifically identified in the parasitic females also contained astacin and CAP domains (see below). Genes that encode astacins and CAP domain-containing proteins are highly expanded in the *S. venezuelensis* genome as well as the genomes of three other *Strongyloides* species, comprising large gene families that contain more than 300 and 200 genes, respectively [21]. Many of these genes have been shown to be differentially expressed between nematode stages [21, 38], which was supported by the findings of this study and suggests functional divergence of these genes in the expanded gene family.

### Parasitic female hydro-soluble proteins

Of the 160 proteins that were identified in the parasitic female hydro-soluble samples, 73 were specific to this group (Fig. 2). These included a wide range of Pfam domains, such as histones (CENP-T), 14-3-3, proisomerase, acetylcholine esterase (COesterase) and ubiquitin (Fig. 3b, Additional file 3: Table S2), among which histones (CENP-T), COesterase and ubiquitin were specific to the parasitic female hydro-soluble sample group (Fig. 3b). This group also contained many proteins of unknown function, with 25 of the 73 proteins not having any GO annotations and showing no similarity to any other proteins or only to other proteins of unknown function. This is significantly higher than the number of unknown proteins in the iL3 samples (70/350 proteins, Chi-square test: *χ*^2^ = 6.25, *df* = 1, *P* = 0.012) or in the entire genome (3145/14048 proteins, Chi-square test: *χ*^2^ = 15.396, *df* = 1, *P* < 0.0001), suggesting that many novel proteins are involved in the establishment of parasitism in parasitic females.

Intriguingly, two of the proteins that had high emPAI values (SVEN_0025100.1 and SVEN_0615900.1) were histone family proteins, which are generally found in the nucleolus of eukaryotic cells. Several other proteins that were identified in this sample group were also somatic or nucleolus proteins, such as actin, ribosomal proteins, elongation initiation factor and cytochrome C, although these had relatively low emPAI values (Additional file 3: Table S2). These results suggest that many proteins are secreted by parasitic females of this species *via* non-classical secretion pathways.

### Parasitic female hydro-insoluble proteins

Most of the proteins that were identified in the parasitic female hydro-insoluble samples were sample-group specific (33 out of a total of 46 proteins; Fig. 2). Six of the 20 most abundant proteins were trypsin inhibitor-like (TIL) domain-containing proteins (Fig. 3c, Additional file 4: Table S3). These six proteins comprised 28% of the total amount (emPAI) in the hydro-insoluble sample, suggesting that TIL proteins are one of the main protein components of the adhesives that are produced by parasitic females (see below for further discussion). Peptidases (astacin, M13 and S10) and SCP/TAPS (CAP-domain) proteins were also identified in this sample group.

### Shared proteins between iL3s and parasitic females

A total of 86 proteins were common to iL3s and parasitic females. These included protein families that have previously been reported as being involved in host-parasite interactions. For instance, enolases had high emPAI values in both iL3s and parasitic females in this study and have been shown to be involved in infection and host immune suppression in the entomopathogenic nematode *Steinernema glaseri* [39]; triosephosphate isomerases have been found to be the most abundant secreted proteins of *Brugia malayi* adults and are involved in microfilalia production [40]; nucleoside diphosphate kinase A is thought to play a role in modulating host cell function in *Trichinella spiralis* [41]; transthyretin [42] and 14-3-3 protein families [43], which have been identified in the E/S components of other helminths, including *S. ratti* [15], *Ascaris suum* [17], *Schistosoma* spp. [44] and *Echinococcus* spp. [45], are believed to have immunogenic roles; nematode fatty acid and retinol binding proteins are thought to be involved in the evasion of primary host plant defence systems in the potato cyst nematode *Globodera pallida* [46]. The identification of these proteins in the E/S components of both iL3s and parasitic females suggests that they play a fundamental role in the parasite-host interaction.

### Comparison of protein and gene expression levels

**Fig. 4:**
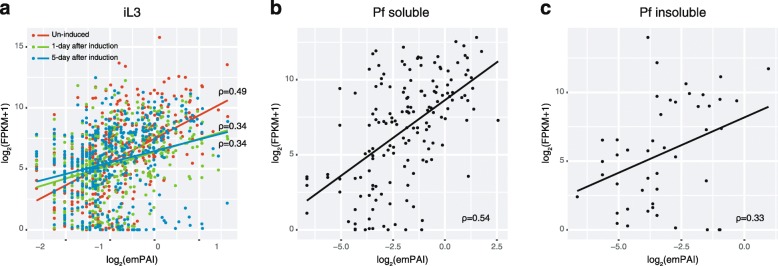
Correlations between the protein and RNA expression levels in infective third-stage larvae (iL3s) (**a**), the parasitic female (Pf) soluble fraction (**b**) and the Pf insoluble fraction of *Strongyloides venezuelensis* (**c**). Protein levels were expressed as exponentially modified protein abundance index (emPAI) values, while RNA expression levels are shown as fragments per kilobase of exon per million reads mapped (FPKM) values. Protein amounts of iL3 were compared with RNA expression levels at three different time points (uninduced and 1 or 5 days after induction)

To examine the relationship between protein and gene expression levels, we compared protein emPAI values with their corresponding gene RNAseq FPKM values (Fig. 4). In parasitic females, the hydro-soluble sample was more strongly correlated with RNAseq FPKM than the hydro-insoluble sample (Correlation: Spearman’s ρ = 0.54, *P* < 0.0001 and 0.33, *P* = 0.02; Fig. 4b, c). In iL3 nematodes, the emPAI of samples obtained pre-induction was more strongly correlated with RNAseq FPKM than those obtained 1 or 5 days post-induction (Correlation: Spearman’s ρ = 0.49, 0.34 and 0.34, respectively, all *P* < 0.0001; Fig. 4a), despite the fact that proteins were obtained by a 1.5 days induced condition (DMEM-incubation). These results suggest that the genes that encode the secreted proteins have already been expressed before induction. Similarly, Maruyama et al. [31] reported that homogenates of iL3 worms obtained before DMEM-incubation showed a high peptidase activity, whereas those obtained after 3 h of DMEM-incubation showed only very low peptidase activity. Together, these findings indicate that high amounts of proteins (or mRNA) for secretion are accumulated in the bodies of pre-infection iL3s and are released immediately after they meet the host, which probably makes it possible for the nematodes to successfully penetrate the skin and invade the host’s body.

### Histones

We identified seven histone proteins [four H2A, one H3 and two H4 (or CENP-T)] in the parasitic female hydro-soluble samples (Additional file 3: Table S2). Histones are found in the nuclei of all eukaryotic cells, where they play a role in packaging DNA into a structural unit known as a nucleosome or chromatin [47]. Consequently, they are not usually secreted outside the cells. We also identified such ‘somatic’ proteins in the secretions of parasitic females (Additional file 3: Table S2), as mentioned above. Although these can be detected from ruptured cells by mechanical or physiological wounding in the sample, the four H2A and two H4 histones had emPAI values of up to 2.83 and 8.06, respectively, suggesting that they were unlikely from wounded or broken cells but likely secreted intentionally by the parasite. It has recently been shown that histones, particularly H2A and H4, have antimicrobial properties that are linked to innate defence in the human intestine [48]. Therefore, the histones that were identified in the present study may be used by the parasite to modify and/or maintain the gut microbiota of the host to produce a beneficial environment.

### TIL domain-containing proteins

**Fig. 5:**
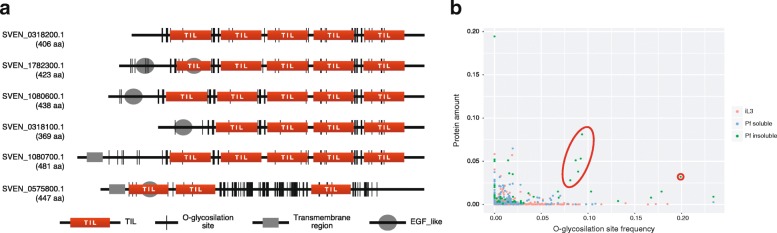
Structural overview of *Strongyloides venezuelensis* trypsin inhibitor-like (TIL) domain-containing proteins (Sv-TILs). **a** All Sv-TILs except SVEN_075800.1 contained four or five tandemly repeated TIL domains. EGF domains were found in SVE_0575800.1, SVE_1080600.1 and SVE_1782300.1. All proteins contained a large number of O-glycosylation sites. Numbers in parentheses represent the protein lengths in amino acids. **b** O-glycosylation site frequency in the proteins identified in the infective larva (iL3) and parasitic female (Pf) samples. Sv-TILs are enclosed by red circles

The adhesive substances that are secreted by *S. venezuelensis* likely play important roles in the infection process in the host’s intestine [20]. They appear to be used by the parasite for attachment to the intestinal epithelium and in the construction of the walls of worm tunnels in the mucosal epithelial layer in which they lodge themselves [20]. As mentioned previously, we found that TIL domain-containing proteins were one of the main components of the adhesive proteins in *S. venezuelensis* (Additional file 4: Table S3). The six *S. venezuelensis* TIL domain-containing proteins (Sv-TILs) that were identified had lengths ranging from 369 to 481 amino acids and five of them had similar structures, consisting of four or five TIL domains (Fig. 5a). Like other typical TIL domains [49], all of the TIL domains in the six proteins had 48 to 58 amino acids and all except one contained 10 cysteine residues, the exception being SVEN_1782300, which had 8 cysteine residues in one domain.

TIL domains are widely distributed in nematodes, arthropods, chordates and echinoderms [50]. In nematodes, it has been suggested that TIL proteins are used to suppress exogenous host proteases [51], facilitate feeding (i.e. anticoagulant function) [52, 53], immunomodulate the host response [54, 55] and control protease function during development and reproduction [56]. Previous comparative studies of the genomes and transcriptomes of *Strongyloides* species have revealed that TIL proteins are one of the eight most important families that are involved in parasitism in *S. ratti/S. stercolaris* [21] and *S. venezuelensis* [38]. We also found that three Sv-TILs (SVE_0575800.1, SVE_1080600.1 and SVE_1782300.1) contained one or two EGF-like domains using SMART protein domain search (Fig. 5a), which are also present in a secreted TIL in *S. ratti* [57]. EGF-like domains are found in a wide variety of proteins, such as growth factors, lipoprotein receptors, selectins, clotting factors and extracellular matrix proteins. The domains occur in the extracellular portion of transmembrane proteins or in secreted proteins and may engage in protein-protein interactions [58, 59]. In some cases, they are also involved in the appropriate exposure of other domains [60] or the formation of complexes [61], which could also be the case for the Sv-TILs.

We found that the Sv-TILs had a large number of O-glycosylation sites (*n* = 33–89) compared with the other secreted proteins (Fig. 5b). Glycosylation is also corroborated by the major band smearing that was observed in the SDS-PAGE gel and the higher molecular mass of Sv-TILs than the theoretical value (45–90 kDa in the gel *vs* 42–54 kDa theoretical value) (Fig. 1). O-linked glycan is also referred to as ‘mucin-type’ glycan and is known to give proteins their adhesive properties. Maruyama & Nawa [19] found that the insoluble secretions of *S. venezuelensis* are rich in carbohydrates, particularly mannose and N-acetyl galactosamine, but are devoid of sialic acid. Furthermore, the adhesive secretions that are produced by the parasitic females bind to sulphated carbohydrates [62]. Therefore, sulphated mucins (e.g. chondroitin sulphate proteoglycan, which is produced by mast cells) are primary candidate molecules for the expulsion of *Strongyloides* adult worms [63, 64].

### Comparison of the secretomes of *S. venezuelensis* and *S. ratti*

Genome and transcriptome comparison of four *Strongyloides* species, including the two rodent parasites *S. venezuelensis* and *S. ratti*, identified the presence of key protein-coding gene families that have a putative role in parasitism in the genus as well as the subclade or species [21, 38]. Comparison of the secretomes of the two rodent parasites is also interesting. A total of 580 proteins were identified in the secretome of *S. ratti* parasitic females [15, 21] and the main protein families were also identified in *S. venezuelensis* (Fig. 3 and Additional file 5: Table S6), despite different methods being used to identify the proteins. For example, Pro_isomerase, transthyretin (TTR-52), COesterase and thioredoxin had high emPAI values in both species. The identification of ‘somatic proteins’, such as actins, histones and elongation factors, in the secretions of both species supports the hypothesis that many proteins are secreted *via* non-classical secretion pathways in *Strongyloides* parasitic females, for example *via* exosome-like vesicles as observed in other gastrointestinal nematodes *Heligmosomoides polygyrus* [65], *Nippostrongylus brasiliensis* [66] and *Trichuris muris* [67].

There were differences, however, in the amounts of peptidase and CAP protein families, with much higher amounts being detected in *S. ratti* than *S. venezuelensis.* Protein families that were uniquely identified as being present at a high amount in *S. venezuelensis* included cytochrome P450 and rhodaneses (Additional file 3: Table S2), whereas those in *S. ratti* included serine peptidases (Peptidase_S9), superoxide dismutases (Sod_Cu) and heat-shock protein 20 (HSP20) (Additional file 1: Figure S2). This may reflect the different parasitism strategies used by the two species such as the difference of within-host microhabitats – villi for *S. venezuelensis* and crypt for *S. ratti* [8], potentially indicating independent adaptation, although we cannot exclude the possibility that this was caused by differences in methodology.

## Conclusions

Excreted and secreted proteins play important roles in parasitic nematodes. In this study, we identified 546 E/S proteins from two distinct infective stages of the intestinal parasitic nematode *S. venezuelensis*. Functional annotation of these proteins and comparisons between the stages and with other species of nematodes indicated that a large number of proteins with different functions participate in establishing parasitism in this species. Our protein identification results will provide a valuable resource for future investigations of the molecular mechanism that underlies host-parasite interactions in this nematode.

## Additional files

Additional file 1: **Figure S1.** Sodium dodecyl sulphate polyacrylamide gel electrophoresis (SDS-PAGE) gel images of excretory/secretory (E/S) proteins from infective third-stage larvae (iL3s) and parasitic females (Pfs) of *Strongyloides venezuelensis*. iL3 proteins were collected from DMEM with/without proteinase inhibitors. Pf proteins were collected from secretions from worms incubated at 37 °C in PBS with or without proteinase inhibitors or at 4 °C without proteinase inhibitors. **Figure S2.** Stoma structure of infective third-stage larvae (iL3s) of *Strongyloides venezuelensis* **a** prior to induction, **b** 36 h post-induction with Dulbecco’s modified Eagle medium (DMEM) at 37 °C, **c** 36 h post-induction with DMEM at 37 °C with proteinase inhibitors and **d** in a nematode isolated from a rat’s (host’s) lung. *Scale-bar*: 20 μm. **Table S4.** Enriched gene ontology (GO) terms for the infective third-stage larva (iL3) samples. **Table S5.** Enriched gene ontology (GO) terms for the parasitic female soluble samples. (PDF 749 kb)

Additional file 2: **Table S1.** List of proteins identified in the *Strongyloides venezuelensis* infective third-stage larva (iL3) excretory/secretory (E/S) samples. (XLSX 80 kb)

Additional file 3: **Table S2.** List of proteins identified in the *Strongyloides venezuelensis* parasitic female excretory/secretory (E/S) soluble fraction. (XLSX 36 kb)

Additional file 4: **Table S3.** List of proteins identified in the *Strongyloides venezuelensis* parasitic female excretory/secretory (E/S) insoluble fraction. (XLSX 18 kb)

Additional file 5: **Table S6.** Pfam protein domains identified in the secretome of *Strongyloides ratti*. Protein identification data were obtained from Hunt et al. [21]. The sum of the exponentially modified protein abundance index (emPAI) values of identified proteins was calculated for each Pfam domain. (XLSX 19 kb)

## Abbreviations

E/S: Excretory/secretory
iL3: Infective third-stage larva
Pf: Parasitic female
TIL: Trypsin inhibitor-like
