# Supplementary material for: Secretome analysis of Strongyloides venezuelensis parasitic stages reveals that soluble and insoluble proteins are involved in its parasitism
Source: Parasit Vectors. 2019 Jan 9;12:21. doi: 10.1186/s13071-018-3266-x (PMC6327390; doi:10.1186/s13071-018-3266-x)
Supplement: Supplementary file 1 — Figure S1. Sodium dodecyl sulphate polyacrylamide gel electrophoresis (SDS-PAGE) gel images of excretory/secretory (E/S) proteins from infective third-stage larvae (iL3s) and parasitic females (Pfs) of Strongyloides venezuelensis. iL3 proteins were collected from DMEM with/without proteinase inhibitors. Pf proteins were collected from secretions from worms incubated at 37 °C in PBS with or without proteinase inhibitors or at 4 °C without proteinase inhibitors. Figure S2. Stoma structure of infective third-stage larvae (iL3s) of Strongyloides venezuelensis a prior to induction, b 36 h post-induction with Dulbecco’s modified Eagle medium (DMEM) at 37 °C, c 36 h post-induction with DMEM at 37 °C with proteinase inhibitors and d in a nematode isolated from a rat’s (host’s) lung. Scale-bar: 20 μm. Table S4. Enriched gene ontology (GO) terms for the infective third-stage larva (iL3) samples. Table S5. Enriched gene ontology (GO) terms for the parasitic female soluble samples. (PDF 749 kb) [file 13071_2018_3266_MOESM1_ESM.pdf]

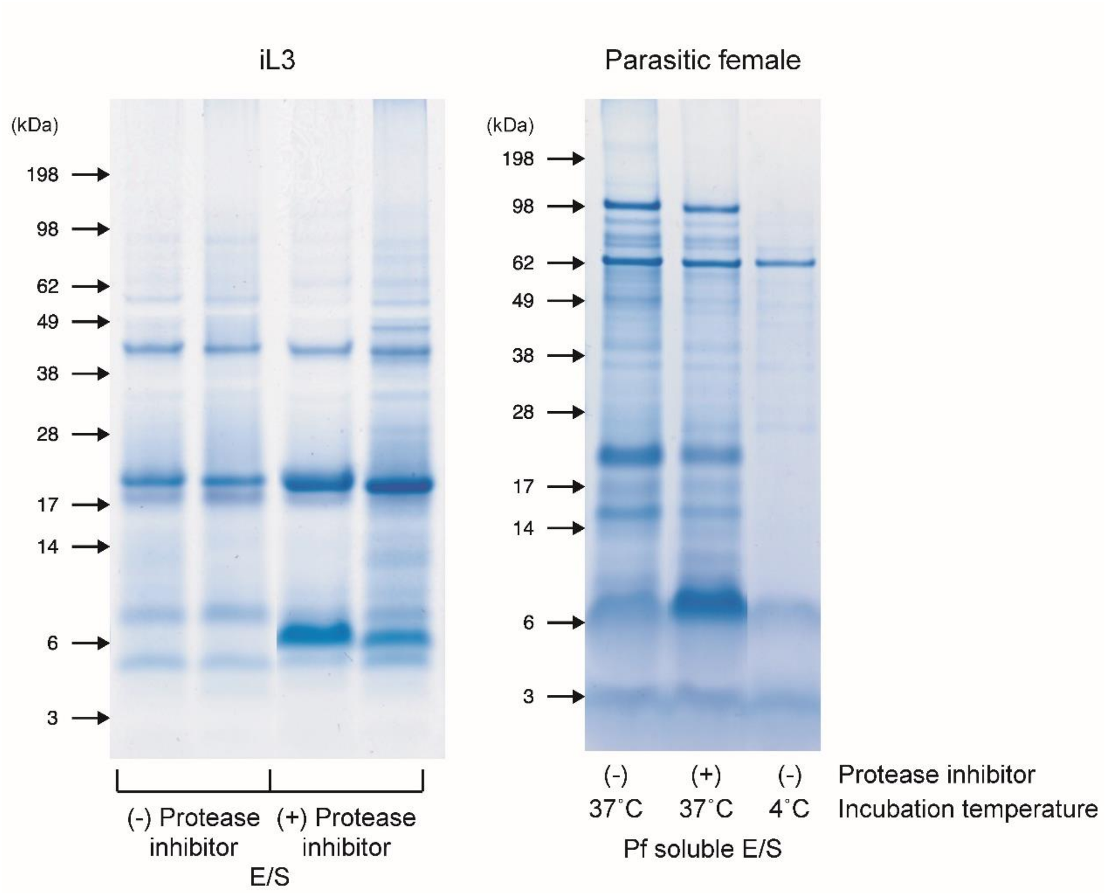

Figure S1. Sodium dodecyl sulphate polyacrylamide gel electrophoresis (SDS-PAGE) gel images of excretory/secretory (E/S) proteins from infective third-stage larvae (iL3s) and parasitic females (Pfs) of *Strongyloides venezuelensis*. iL3 proteins were collected from DMEM with/without proteinase inhibitors. Pf proteins were collected from secretions from worms incubated at 37°C in PBS with or without proteinase inhibitors or at 4°C without proteinase inhibitors.

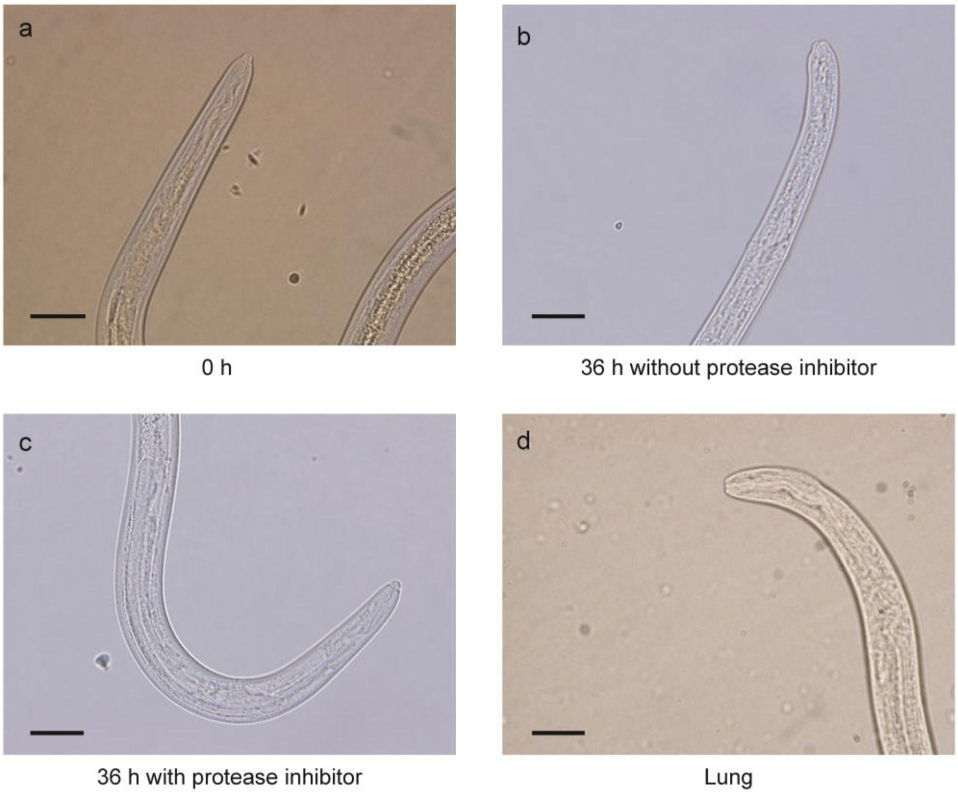

Figure S2. Stoma structure of infective third-stage larvae (iL3s) of *Strongyloides venezuelensis* (a) prior to induction, (b) 36 h post-induction with Dulbecco's Modified Eagle Medium (DMEM) at 37°C, (c) 36 h post-induction with DMEM at 37°C with proteinase inhibitors and (d) in a nematode isolated from a rat's (host's) lung. Scale bar = 20 μm.

Table S4. Enriched gene ontology (GO) terms for the infective third-stage larva (iL3) samples.

GO-ID	Term	Category*	FDR	P-Value	#Test	#Ref	#notAnnotTest	#notAnnotRef	Over/Under
GO:0009792	embryo development ending in birth or egg hatching	P	7.79E-19	2.36E-22	61	373	307	10286	over
GO:0002119	nematode larval development	P	3.10E-13	6.11E-16	52	385	316	10274	over
GO:0008340	determination of adult lifespan	P	6.60E-13	1.60E-15	31	131	337	10528	over
GO:0004298	threonine-type endopeptidase activity	F	4.90E-11	1.86E-13	11	5	357	10654	over
GO:0010499	proteasomal ubiquitin-independent protein catabolic process	P	1.01E-10	3.98E-13	10	3	358	10656	over
GO:0006096	glycolysis	P	4.34E-09	2.50E-11	11	11	357	10648	over
GO:0040011	locomotion	P	2.60E-08	1.73E-10	42	380	326	10279	over
GO:0006099	tricarboxylic acid cycle	P	5.70E-08	3.98E-10	11	16	357	10643	over
GO:0043161	proteasomal ubiquitin-dependent protein catabolic process	P	4.22E-07	3.45E-09	13	35	355	10624	over
GO:0006898	receptor-mediated endocytosis	P	5.36E-07	4.55E-09	19	93	349	10566	over
GO:0019773	proteasome core complex, alpha-subunit complex	C	1.03E-06	9.03E-09	6	1	362	10658	over
GO:0030170	pyridoxal phosphate binding	F	7.96E-06	8.32E-08	11	31	357	10628	over
GO:0040007	growth	P	3.74E-05	4.30E-07	20	142	348	10517	over
GO:0000287	magnesium ion binding	F	1.39E-04	1.90E-06	11	45	357	10614	over
GO:0016616	oxidoreductase activity, acting on the CH-OH group of donors, NAD or NADP as acceptor	F	1.83E-04	2.61E-06	10	37	358	10622	over
GO:0005977	glycogen metabolic process	P	2.11E-04	3.17E-06	6	8	362	10651	over
GO:0048871	multicellular organismal homeostasis	P	3.81E-04	5.94E-06	4	1	364	10658	over
GO:0051015	actin filament binding	F	4.49E-04	7.43E-06	8	24	360	10635	over
GO:0005759	mitochondrial matrix	C	6.71E-04	1.16E-05	10	45	358	10614	over
GO:0033178	proton-transporting two-sector ATPase complex, catalytic domain	C	6.71E-04	1.20E-05	6	11	362	10648	over
GO:0005882	intermediate filament	C	6.71E-04	1.20E-05	6	11	362	10648	over
GO:0050662	coenzyme binding	F	7.06E-04	1.27E-05	15	107	353	10552	over
GO:0004029	aldehyde dehydrogenase (NAD) activity	F	8.59E-04	1.58E-05	5	6	363	10653	over
GO:0005504	fatty acid binding	F	9.16E-04	1.74E-05	4	2	364	10657	over
GO:0071688	striated muscle myosin thick filament assembly	P	9.17E-04	1.75E-05	6	12	362	10647	over
GO:0018996	molting cycle, collagen and cuticulin-based cuticle	P	1.14E-03	2.27E-05	14	99	354	10560	over
GO:0010171	body morphogenesis	P	1.14E-03	2.27E-05	14	99	354	10560	over
GO:0006915	apoptotic process	P	1.49E-03	3.10E-05	16	131	352	10528	over
GO:0052548	regulation of endopeptidase activity	P	1.65E-03	3.47E-05	9	41	359	10618	over
GO:0006457	protein folding	P	1.65E-03	3.55E-05	11	64	357	10595	over
GO:0040035	hermaphrodite genitalia development	P	1.65E-03	3.64E-05	13	90	355	10569	over
GO:0016853	isomerase activity	F	1.65E-03	3.64E-05	13	90	355	10569	over

GO:0006570	tyrosine metabolic process	P	1.65E-03	3.69E-05	3	0	365	10659	over
GO:0006559	L-phenylalanine catabolic process	P	1.65E-03	3.69E-05	3	0	365	10659	over
GO:0004054	arginine kinase activity	F	1.65E-03	3.69E-05	3	0	365	10659	over
GO:0016668	oxidoreductase activity, acting on a sulfur group of donors, NAD(P) as acceptor	F	1.73E-03	3.94E-05	4	3	364	10656	over
GO:0072593	reactive oxygen species metabolic process	P	1.73E-03	3.94E-05	4	3	364	10656	over
GO:0016615	malate dehydrogenase activity	F	3.16E-03	7.68E-05	4	4	364	10655	over
GO:0006108	malate metabolic process	P	3.16E-03	7.68E-05	4	4	364	10655	over
GO:0016829	lyase activity	F	3.16E-03	7.72E-05	15	127	353	10532	over
GO:0044769	ATPase activity, coupled to transmembrane movement of ions, rotational mechanism	F	3.37E-03	8.27E-05	6	17	362	10642	over
GO:0005865	striated muscle thin filament	C	5.13E-03	1.30E-04	5	11	363	10648	over
GO:0048471	perinuclear region of cytoplasm	C	5.32E-03	1.37E-04	6	19	362	10640	over
GO:0034614	cellular response to reactive oxygen species	P	5.36E-03	1.44E-04	3	1	365	10658	over
GO:0005501	retinoid binding	F	5.36E-03	1.44E-04	3	1	365	10658	over
GO:0008184	glycogen phosphorylase activity	F	5.36E-03	1.44E-04	3	1	365	10658	over
GO:0004784	superoxide dismutase activity	F	5.36E-03	1.44E-04	3	1	365	10658	over
GO:0048609	multicellular organismal reproductive process	P	6.90E-03	1.91E-04	19	206	349	10453	over
GO:0061134	peptidase regulator activity	F	7.12E-03	1.98E-04	9	53	359	10606	over
GO:0046034	ATP metabolic process	P	7.64E-03	2.16E-04	6	21	362	10638	over
GO:0015991	ATP hydrolysis coupled proton transport	P	7.64E-03	2.16E-04	6	21	362	10638	over
GO:0016051	carbohydrate biosynthetic process	P	7.64E-03	2.16E-04	6	21	362	10638	over
GO:0045454	cell redox homeostasis	P	7.87E-03	2.25E-04	7	31	361	10628	over
GO:0008483	transaminase activity	F	8.28E-03	2.41E-04	5	13	363	10646	over
GO:0044706	multi-multicellular organism process	P	8.48E-03	2.48E-04	13	111	355	10548	over
GO:0019098	reproductive behavior	P	9.84E-03	2.91E-04	13	113	355	10546	over

* P: biological process, C: cellular component and F: molecular function

Table S5. Enriched gene ontology (GO) terms for the parasitic female soluble samples.

GO-ID	Term	Category*	FDR	P-Value	#Test	#Ref	#notAnnotTest	#notAnnotRef	Over/Under
GO:0006096	glycolysis	P	3.76E-07	5.71E-11	8	14	116	10889	over
GO:0006457	protein folding	P	8.20E-05	1.44E-07	9	66	115	10837	over
GO:0000786	nucleosome	C	8.20E-05	1.49E-07	7	30	117	10873	over
GO:0046982	protein heterodimerization activity	F	1.09E-03	2.82E-06	7	49	117	10854	over
GO:0016853	isomerase activity	F	6.89E-03	1.98E-05	8	95	116	10808	over
GO:0005504	fatty acid binding	F	8.22E-03	2.71E-05	3	3	121	10900	over
GO:0009792	embryo development ending in birth or egg hatching	P	8.22E-03	2.74E-05	16	418	108	10485	over
GO:0040018	positive regulation of multicellular organism growth	P	8.97E-03	3.67E-05	6	50	118	10853	over
GO:0071688	striated muscle myosin thick filament assembly	P	9.07E-03	4.13E-05	4	14	120	10889	over
GO:0055114	oxidation-reduction process	P	9.11E-03	4.44E-05	17	485	107	10418	over
GO:0006334	nucleosome assembly	P	9.11E-03	5.18E-05	4	15	120	10888	over

* P: biological process, C: cellular component and F: molecular function

Table S1 (in a separate file). List of proteins identified in the *Strongyloides venezuelensis* infective third-stage larva (iL3) excretory/secretory (E/S) samples.

Table S2 (in a separate file). List of proteins identified in the *Strongyloides venezuelensis* parasitic female excretory/secretory (E/S) soluble fraction.

Table S3 (in a separate file). List of proteins identified in the *Strongyloides venezuelensis* parasitic female excretory/secretory (E/S) insoluble fraction.

Table S6 (in a separate file). Pfam protein domains identified in the secretome of *Strongyloides ratti*. Protein identification data were obtained from Hunt et al. (2016). The sum of the exponentially modified protein abundance index (emPAI) values of identified proteins was calculated for each Pfam domain
